# Mediated effects of a randomised control trial for a text messaging smoking cessation intervention for online help-seekers and primary care visitors

**DOI:** 10.1186/s12889-024-19273-4

**Published:** 2024-07-09

**Authors:** Joel Crawford, Jenny Blomqvist, Katarina Ulfsdotter Gunnarsson, Preben Bendtsen, Marcus Bendtsen

**Affiliations:** 1 Department of Health, Medicine and Caring Sciences, Linköping University, 581 83 Linköping, Sweden; 2 Department of Medical Specialist, Motala, Sweden

**Keywords:** Smoking cessation intervention, Digital intervention, Mediator, Public health, Randomised controlled trial

## Abstract

**Background and Aims:**

Digital smoking cessation interventions have been shown to be effective in helping individuals achieve prolonged smoking abstinence. Nonetheless, the mechanisms that drive such effects are unclear. The current study aimed to estimate a digital smoking cessation intervention's natural direct and indirect effects.

**Methods:**

This secondary analysis of mediated effects uses data from a randomised controlled trial which included participants who smoked at least one cigarette a week, had access to a mobile phone, and were 18 years or older. The comparator was existing smoking cessation support available to all members of the Swedish public. Primary outcomes were prolonged smoking abstinence and point prevalence of smoking abstinence, measured at 3- and 6-months post-randomisation. A counterfactual framework was used to estimate three hypothesised mediators of the intervention's effects: importance, knowledge of how to change (know-how), and confidence.

**Results:**

Between 18/09/20 and 16/06/22, 1012 participants were randomised. The intervention led to improved confidence and know-how, which both partially mediated the effects of the digital intervention on smoking abstinence at 3- and 6 months post-randomisation.

**Conclusions:**

A digital smoking cessation intervention was found to partially affect smoking abstinence by improving individuals’ confidence in their ability to quit smoking and developing knowledge on how to quit. Face-value single-item mediator measures, lack of blinding, and attrition limit the study. Future studies should address these limitations and assess additional mechanisms mediating intervention effects.

**Trial registration:**

ISRCTN13455271.

**Supplementary Information:**

The online version contains supplementary material available at 10.1186/s12889-024-19273-4.

## Background

In Sweden, smoking prevalence within the adult population has steadily decreased over the last two decades. Despite this, smoking remains a leading cause of mortality and morbidity in Sweden, being attributed to approximately 12,000 deaths and 100,000 cases of smoking-related illnesses per year [1]. Smoking also contributes to health inequalities in Sweden, as evidence suggests an inverse relationship between socio-economic status and smoking prevalence [2]. In response, digital interventions have been developed to provide smoking cessation support to those in society who may benefit. These interventions are low-cost and typically include the usage of web pages, mobile phone apps, and text messages to deliver support for behaviour change [3].

Trials of text message-based smoking cessation support have shown that these interventions can successfully increase abstinence across various populations and nations [4]. Within Sweden, trials of text messaging interventions have been conducted using the NEXit (nicotine exit) protocol (see Bendtsen et al. [3] for details). Trials using the protocol have successfully shown prolonged abstinence in university and high school students [5–7]. Recently, a NEXit trial [8] investigated if providing support via text messaging was successful in increasing smoking abstinence within the Swedish general population (i.e., older adults who may have been smoking for a longer time and have had failed cessation attempts using existing support). The results indicated that the odds of prolonged abstinence and point prevalence of abstinence among those given access to the intervention in addition to publicly available support was higher at 3- and 6 months post-randomisation, compared to those who received publicly available support only. The odds ratio (OR) of prolonged abstinence at 3 months was 2.15 (95% compatibility interval [CoI] = 1.51; 3.06, probability of effect [POE] > 99.9%, *p* < 0.0001); and at six months, the OR of prolonged abstinence was 2.38 (95% CoI = 1.62; 3.57, POE > 99.9%, *p* < 0.0001). For point prevalence of abstinence, the OR at 3 months was 1.70 (95% CoI = 1.18; 2.44, POE = 99.8%, p = 0.0034), and at 6 months it was 1.49 (95% CoI = 1.03; 2.14, POE = 98.3%, p = 0.0349).

The advice delivered in the intervention was informed by factors essential in eliciting behavioural change: increasing behavioural intention, addressing environmental constraints, and enhancing behavioural skills needed to change [9]. These factors are central to socio-cognitive models of health that have been used extensively in health research over the last 70 years [10]. Techniques that have been shown to be effective in manipulating these factors in relation to smoking cessation typically focus on social support, commitment, problem-solving, self-regulation, and distraction [11]. Pertinent to the current study, we hypothesised that by promoting these actions we could influence three mediators of change: (1) the degree to which participants believe it is important to make a change [12–14]; (2) participants’ knowledge of how to implement actions aimed at smoking abstinence (know-how) [9, 10]; and (3) participants’ confidence in being able to make a change they specified in abstaining from smoking [15]. Table 1 details the intervention content and how they were hypothesised to relate to the behavioural factors and mediators of change.

**Table 1:** Intervention content and the respective behavioural factors and mediators

Intervention content	Behaviour change domain	Mediator	Model of health
Making a public declaration about quitting	Intentions	Importance/Confidence	Theory of Planned behaviour/Protection motivation theory
Asking for social support from family and friends	Intentions/environment	Confidence	Protection motivation theory
Information on the health risks of smoking	Intentions	Importance	Protection motivation theory
Distraction techniques	Skills	Know-how	Theory of Planned behaviour
Tips to avoid weight-gain	Skills	Confidence/Know-how	Theory of Planned behaviour/Protection motivation theory
Tips to cope with cravings	Skills	Confidence/Know-How	Theory of Planned behaviour/Protection motivation theory
Avoidance of smoking triggers	Environment	Confidence	Protection motivation theory
Self-regulation techniques	Skills	Confidence/Know-How	Theory of Planned behaviour
Encouragement and affirmation	Intentions	Confidence	Protection motivation theory

Understanding the mechanisms that drive the observed effects leading to behavioural change can aid the development of more effective future interventions [16]. Therefore, the objective of the current study was to estimate the degree to which importance, know-how, and confidence mediated the causal relationship between the NEXit intervention and smoking abstinence. This study was nested within a trial whose primary objective was to estimate the total effect of the intervention on smoking abstinence [8]. This facilitated the estimation of natural direct and natural indirect effects by including measures of the potential mediating factors at the multiple follow-up intervals.

## Methods

The current study investigated the mediated effects of a smoking cessation intervention and used data from a 2-arm, parallel groups (1:1), randomised controlled trial (RCT). The trial was preregistered on the ISRCTN registry on 03/12/2020 (ISRCTN13455271) and received ethical approval from the Swedish Ethical Review Authority on 16/06/2020 (Dnr 2020–01427). A protocol and statistical analysis plan was published prior to recruitment (Bendtsen et al. 2020). This study report uses the guidelines outlined for mediator analysis reporting in the AGReMA statement [17].

### Setting and participants

The target population was Swedish adults seeking help to stop smoking. Individuals were required to be 18 years or older, smoke at least one cigarette per week (reasoned as the lowest threshold to benefit from the intervention), have access to a mobile phone, and have a good comprehension of Swedish. Participants were recruited in two settings: (1) online advertisements (Google and Facebook), through which individuals clicked on a link and were taken to the study webpage that contained study information and instructions for signing up; and (2) healthcare professionals at 54 participating primary health care units in the south of Sweden advertised the trial to patients through printed media (e.g., leaflets and posters). The printed media contained study information and instructions for signing up. Regardless of setting, participants were required to sign up for the trial by sending a text message to a dedicated telephone number. Within 5 min, participants received a reply containing a hyperlink to study information and an informed consent form. Those who consented were asked to respond to a baseline questionnaire (which was used to assess eligibility). Immediately after completing the baseline questionnaire, eligible participants were randomised to one of two study groups.

### Interventions

The intervention and the control group were given treatment as usual, and neither was restricted from using other publicly available smoking cessation aids. In addition, participants in the intervention group were given access to the text messaging intervention. Treatment as usual for those recruited online was defined as a referral to a national smoking cessation helpline and a national website with general information regarding smoking and health. For those recruited through primary health care units, treatment-as-usual was defined the same as for online participants, with an additional referral to meet with a nurse or smoking cessation counsellor about smoking cessation and health. Two versions of the text messaging intervention were available: a general version and one for those undergoing elective surgery. The elective surgery intervention was allocated to those reporting having elective surgery planned in the next three months. It included additional messages that spoke directly about the impact smoking has on complications and recovery after surgery. Both intervention versions consisted of a 12-week text message program with messages sent daily to their mobile phones (for full details, see the protocol Bendtsen et al. [3]). The messages were informational, provided encouragement, and included prompts for completing specific tasks to enable and sustain change (e.g., disposing of ashtrays, setting a date for quitting). Participants could request additional content by sending a text if they were experiencing cravings or had relapsed.

### Randomisation and blinding

Participants were randomised using a computer-generated random sequence. Participants were stratified according to which of the two intervention versions was appropriate (i.e., general or surgery). Block randomisation was used to ensure an equal number of participants in each group within the stratum, using random block sizes of 2 and 4. Participants were informed about group allocation via text message. Research personnel could not affect the allocation, and all study procedures were automated (except follow-up calls to non-responders), thus preventing subversion of the allocation concealment.

### Sample size

Since this study was nested within an effectiveness trial, where the primary objective was to estimate the intervention's total effect, no power calculation was done specifically for the mediation analyses. The power calculation for the primary objective was done using a Monte Carlo study, the details of which can be found in the protocol [3].

### Outcomes and measures

#### Mediators

Three potential mediating factors were assessed: importance, know-how and confidence. The factors are reflective of the behavioural and control beliefs that drive behaviour as outlined in expectancy-value models of health (e.g., protection-motivation theory – 14; the theory of planned behaviour—12). Importance, or outcome efficacy, is reflective of behavioural beliefs, in which the motivation to change is influenced by the belief of how valuable a desired outcome is [13]. If an individual places great value on an outcome such as smoking cessation, the motivation to change behaviour increases. Know-how, or having knowledge of how to change, is reflective of control beliefs, in which we feel we have the requisite knowledge or skills to achieve an outcome [12]. Confidence is a shorthand for self-efficacy and is further reflective of control beliefs, in which we perceive we have agency in performing the given behaviour [13].

All three mediators were measured at baseline, and 1-, 3-, and 6-months post randomisation. To help reduce the load on participants across the trial period, face-valid, single-item measures were used. These items are based on importance and confidence rules [18], typically used without a fixed time interval, instead focusing on a general assessment in lieu of a specific time frame. The items, which were also used in a previous study of mediators of a digital alcohol intervention [19], are as follows:

**Importance**: “How important is it for you to quit smoking?” (10-point scale ranging from 1 = “Not important” to 10 = “Very important”).

**Know-how**: “How well do you know how to quit smoking?” (10-point scale ranging from 1 = “Not well at all” to 10 = “Very well”).

**Confidence**: “How confident are you that you will be able to quit smoking?” (10-point scale ranging from 1 = “Not at all” to 10 = “Very confident”).

Note that at the 3- and 6-month intervals, “and stay smoke-free” was added to the questions to accommodate those who had already quit.

### Outcomes

The two co-primary outcomes in the parent trial were self-reported prolonged abstinence and point prevalence of smoking abstinence, measured at 3- and 6-months post-randomisation. Prolonged abstinence was defined using the Russell standard [20] of not having smoked more than 5 cigarettes in the past 8 weeks at the 3-month follow-up interval and in the past 5 months at the 6-month follow-up interval (thus allowing for a 4-week grace period from the start of the intervention program). Point prevalence was defined as not smoking any cigarettes during the past 4 weeks at both follow-up intervals [21].

### Follow-up procedure

Follow-ups to collect outcome data at 3- and 6 months post-randomisation were initiated by sending a text message to participants with a hyperlink to the questionnaires. A total of two reminders were sent two days apart to non-responders. If no response was received after the second reminder, a text message was sent asking participants to respond directly to the text message. Finally, if no response was received from the text message, then an attempt to call the participants to collect data was made for primary outcomes (a maximum of 5 call attempts were made). There was also a 1-month follow-up to facilitate the enclosed mediation analyses, where mediator variables alone were measured. The same reminder procedure was used, except no calls were made to collect data.

### Effects of interest

The effects of interest were natural direct and natural indirect effects, as outlined by Pearl [22]. The natural direct effect is the expected change in outcomes in response to moving from the control group to the intervention group, whilst keeping the value of the mediator constant if the move had not occurred. In contrast the indirect effect is the expected change in outcomes in response to remaining in the control group but changing the value of the mediator to the value it would have taken from making the change from the control group to the intervention group. Considering these definitions, the study aimed to estimate:

The natural direct effect of treatment allocation on smoking cessation outcomes at 3- and 6 months post-randomisation,

The natural indirect effect of treatment allocation through mediating factors at 1-month post-randomisation on smoking cessation outcomes at 3 months post-randomisation,

The natural indirect effect of treatment allocation through mediating factors at 3 months post-randomisation on smoking cessation outcomes at 6 months post-randomisation.

**Fig. 1:**
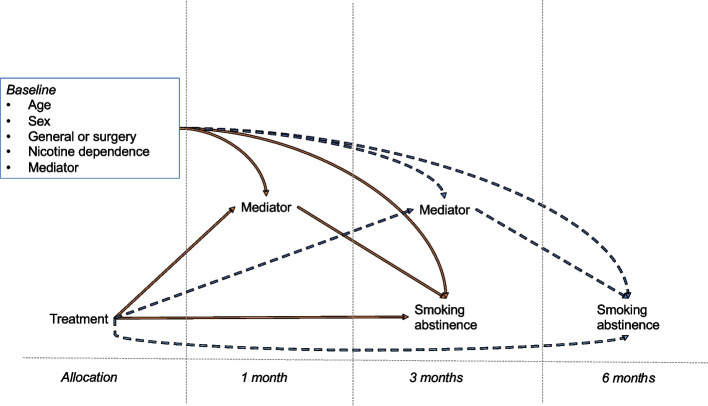
Causal model representing baseline characteristics, treatment, mediators, and outcomes at allocation and follow-up interval

The estimation of these effects was outlined in the trial protocol as a secondary objective of the trial, following the primary total effects of the intervention on smoking cessation (3). Causal models were used to estimate, (1) each individual mediating factor and (2) a single model combining all three mediating factors. Figure 1 depicts the causal model, where the orange model was used for 3-month outcomes and the blue-dashed model for 6-month outcomes. The models were built using the assumption that accounting for baseline characteristics reduces the impact of confounding factors on the mediators and outcomes, however this assumption cannot be tested and should be considered when interpreting findings.

### Statistical methods

Participants were analysed in the groups to which they were randomised (intention-to-treat). Missing data was initially handled by available data analysis, complemented with sensitivity analyses where missing data was imputed using multiple imputation with chained Eqs. [23], please see Appendix A for details. Analyses were done using R version 4.3.1 and CmdStan version 2.30.1.

The analyses were conducted using a counterfactual framework following Pearl's mediation formulas [22]. Logistic regression was used to model smoking abstinence outcomes and linear regression to model mediator variables (which were standardised). Treatment-mediator and treatment-mediator-outcome models were adjusted for sex, age, general or surgery version of intervention, baseline Fagerström test for nicotine dependence score, and baseline values for each respective mediator. Models were estimated using Bayesian inference [24], with standard normal priors for all coefficients representing effects. We will use the medians of posterior distributions as point estimates, which will be presented with 95% compatibility intervals (CI) defined by the 2.5% and 97.5% percentiles of the posterior distribution.

### Attrition analyses

Attrition analyses investigated if responders and non-responders differed systematically with respect to baseline characteristics and among study groups. We used logistic regression with an interaction for group allocation and estimated the models with Bayesian inference [24]. We used Cauchy priors for coefficients with a normal hyperprior for the scale parameter [25] to account for the excessive number of covariates.

The total effect of intervention on smoking cessation outcomes were estimated using data from participants for which mediation data was available. These estimates were compared to estimates of total effect from the primary analyses of the trial which have already been reported [8]. Marked differences between estimates may be indicative of systematic differences between participants with mediation data available and those without.

## Results

**Fig. 2:**
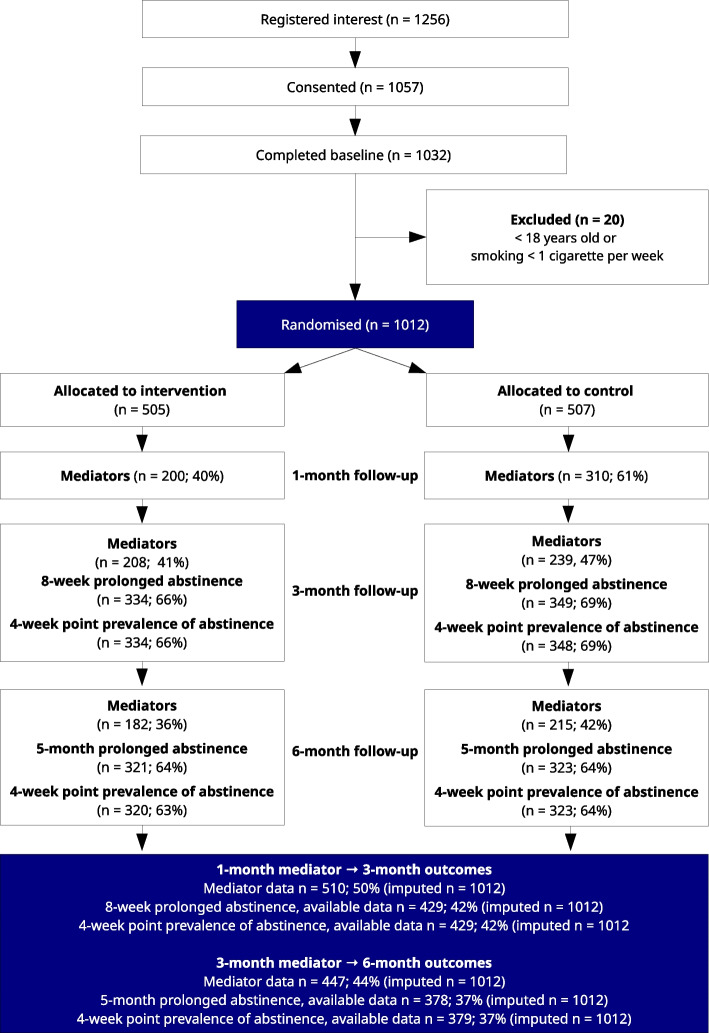
CONSORT flowchart

Between 18/09/2020 and 16/06/2022, a total of 1256 participants registered their interest in the trial, of which 1057 provided informed consent. Eligible were 1012 participants, who were randomised to intervention (*n* = 505) and control (*n* = 507). Participants were predominantly recruited online (95.9%, *n* = 971), and 6.2% (*n* = 63) reported having elective surgery planned in the next three months. Baseline characteristics are reported in Table 2, and a CONSORT flowchart is available in Fig. 2.

**Table 2:** Baseline characteristics of randomised participants

	**Total (*n* = 1012)**	**Intervention (*n* = 505)**	**Control (*n* = 507)**
Woman^a^, n (%)	820 (81)	406 (80.4)	414 (81.7)
Age, mean (SD)	45.4 (14)	45 (13.9)	45.7 (14.1)
Years of smoking, mean (SD)	25.3 (14.6)	24.7 (14.3)	25.9 (14.9)
Cigarettes smoked per week, mean (SD)	101 (46.2)	101.4 (47.3)	100.6 (45.1)
**Use of snus**^b^, ***n* (%)**			
Daily	63 (6.2)	27 (5.3)	36 (7.1)
Weekly or monthly	90 (8.9)	45 (8.9)	45 (8.9)
No	859 (84.9)	433 (85.7)	426 (84.0)
Fagerström test for nicotine dependence, mean (SD)	5 (2.2)	5 (2.2)	5 (2.2)
Number of quit attempts^c^, mean (SD)	7.2 (13.7)	7.0 (12.7)	7.5 (14.6)
**Cessation counselling experience, >*n*(%)**>			
Yes, now	32 (3.2)	13 (2.6)	19 (3.7)
Yes	192 (19.0)	95 (18.8)	97 (19.1)
No	788 (77.9)	397 (78.6)	391 (77.1)
Used quit smoking helpline, *n* (%)	138 (13.6)	73 (14.5)	65 (12.8)
Importance of quitting^d^, mean (SD)	9.4 (1.3)	9.4 (1.3)	9.5 (1.2)
Confidence in ability to quit^d^, mean (SD)	6.2 (2.5)	6.3 (2.5)	6.2 (2.6)
Knowledge of how to quit^d^, mean (SD)	5.5 (2.6)	5.5 (2.7)	5.5 (2.5)

^a^ The baseline questionnaire included a category “Other”, however, it was not chosen by any participant

^b^ Snus is a moist oral tobacco product which is common in Sweden, sometimes translated as *snuff*

^c^ Participants were asked about the lifetime number of quit attempts

^d^ Three single item measures were used to assess importance, confidence, and know-how regarding smoking cessation. Responses ranged from 0 to 10, with 10 representing highest agreement (i.e., very important, very confident, very knowledgeable). The same items were used at follow-up as hypothesised mediators of effects

### Outcomes and estimates

At the 1-month follow-up interval, mediator measures were collected from 50% of participants (intervention group: 40%, control group: 61%). At the 3-month follow-up interval, mediator measures were collected from 44% of participants (intervention group: 41%, control group: 47%). At the 6-month follow-up interval, mediator measures were collected from 39% of participants (intervention group: 36%, control group: 42%). There was evidence that older individuals in both groups were more likely to respond to follow-up of mediator variables at all three intervals. This was also the case for smoking cessation outcomes, as reported in the main trial findings [8]. In addition, there was evidence that among intervention group participants only, those who scored higher on the Fagerström test for nicotine dependence at baseline were more likely to not respond at the 1-month follow-up interval. Furthermore, this association explained entirely the differential follow-up rates between groups at the 1-month follow-up. The association between Fagerström test for nicotine dependence score and response was not found at the subsequent follow-ups at 3- and 6-months. Please see Appendix B for details of the attrition analysis results.

### Effects of intervention on mediator outcomes

**Fig. 3:**
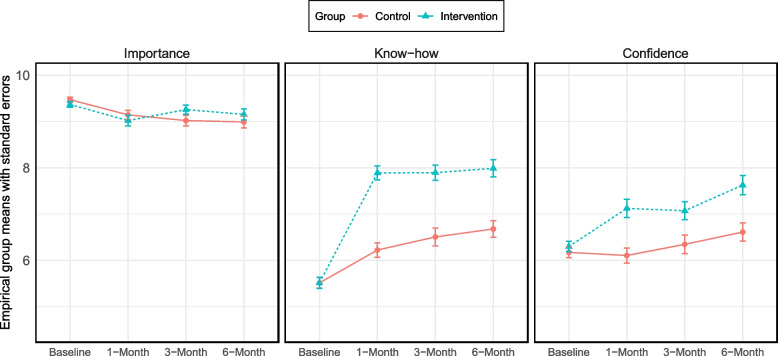
Empirical means and standard errors of mediator measures at baseline, 1-, 3- and 6-months

Figure 3 presents mean and standard errors for mediator measures for each group at baseline and each follow-up interval (non-standardised). Table 3 presents adjusted standardised effect estimates of treatment on mediators for the available data. The results highlight an observed effect on know-how and confidence, however, there was no observed effect of intervention on importance, with both groups reporting a high level of importance at baseline.

**Table 3:** Estimate of adjusted standardised effects of treatment on mediator factors at 1-, 3- and 6 months with available data

	**1-Month**		**3-Month**		**6-Month**	
	**Est. 95% CI**	**Pr.(Est. > 0)**	**Est. 95% CI**	**Pr.(Est. > 0)**	**Est. 95% CI**	**Pr.(Est. > 0)**
**Importance**						
Intervention vs Control	-0.04 (-0.21; 0.12)	70.3%	0.13 (-0.04; 0.3)	93.3%	0.10 (-0.09; 0.29)	84.4%
**Know-how**						
Intervention vs Control	0.55 (0.4; 0.71)	> 99.9%	0.47 (0.3; 0.64)	> 99.9%	0.47 (0.29; 0.65)	> 99.9%
**Confidence**						
Intervention vs Control	0.30 (0.14; 0.46)	> 99.9%	0.23 (0.06; 0.4)	99.6%	0.32 (0.14; 0.5)	> 99.9%

*Abbreviations:* Est. Median of the marginal posterior distribution of adjusted standardised effects, *CI* Compatibility interval (defined by the 2.5% and 97.5% percentiles of the posterior distribution), *Pr* Posterior probability

### Natural direct and natural indirect effects

Estimates of natural direct and natural indirect effects of treatment allocation on 8-week and 5-month prolonged abstinence expressed as ORs, are presented in Table 4. Table 5 presents the estimates of natural direct and natural indirect effects of treatment allocation on 4-week point prevalence of prolonged abstinence. The direct effects were attenuated towards the null in the imputed data analyses, but the indirect effects were similar. Overall, the findings regarding mediation from results produced using available data were no different than using imputed data; therefore, available data analyses are presented here, and imputed data analyses can be found in Appendix A.

**Table 4:** Natural direct and natural indirect effects on 8-week/5-month prolonged abstinence

	**1-month mediator—> 3-month outcome**		**3-month mediator—> 6-month outcome**	
	**Est. OR 95% CI**	**Pr. (OR > 1)**	**Est. OR 95% CI**	**Pr. (OR > 1)**
**Full model (All three mediators)**				
Natural indirect effect	1.81 (1.41; 2.45)	> 99.9%	1.39 (1.14; 1.77)	99.9%
Natural direct effect	1.95 (1.19; 3.15)	99.6%	2.59 (1.56; 4.36)	> 99.9%
**Importance**				
Natural indirect effect	0.98 (0.89; 1.06)	70.4%	1.08 (0.97; 1.28)	92.6%
Natural direct effect	2.98 (1.92; 4.64)	> 99.9%	3.03 (1.90; 4.89)	> 99.9%
**Know-how**				
Natural indirect effect	1.55 (1.28; 1.95)	> 99.9%	1.31 (1.12; 1.61)	> 99.9%
Natural direct effect	2.17 (1.36; 3.44)	> 99.9%	2.43 (1.51; 3.99)	> 99.9%
**Confidence**				
Natural indirect effect	1.41 (1.17; 1.80)	> 99.9%	1.20 (1.04; 1.44)	99.6%
Natural direct effect	2.30 (1.44; 3.72)	> 99.9%	2.81 (1.73; 4.61)	> 99.9%

*Abbreviations:* Est. *OR* Median of the marginal posterior distribution of adjusted odds ratios (OR), *CI* Compatibility interval (defined by the 2.5% and 97.5% percentiles of the posterior distribution), *Pr* Posterior probability

**Table 5:** Natural direct and natural indirect effects on 4-week point prevalence

	**1-month mediator—> 3-month outcome**		**3-month mediator—> 6-month outcome**	
	**Est. OR 95% CI**	**Pr. (OR > 1)**	**Est. OR 95% CI**	**Pr. (OR > 1)**
**Full model (All three mediators)**				
Natural indirect effect	1.46 (1.20; 1.85)	> 99.9%	1.36 (1.14; 1.68)	> 99.9%
Natural direct effect	1.97 (1.24; 3.22)	99.7%	1.22 (0.75; 2.01)	79.2%
**Importance**				
Natural indirect effect	0.99 (0.91; 1.05)	70.4%	1.07 (0.98; 1.22)	92.7%
Natural direct effect	2.75 (1.80; 4.27)	> 99.9%	1.55 (0.98; 2.42)	96.8%
**Know-how**				
Natural indirect effect	1.39 (1.18; 1.71)	> 99.9%	1.29 (1.12; 1.57)	> 99.9%
Natural direct effect	2.09 (1.34; 3.35)	> 99.9%	1.24 (0.77; 1.99)	81.8%
**Confidence**				
Natural indirect effect	1.22 (1.09; 1.44)	> 99.9%	1.16 (1.04; 1.36)	99.6%
Natural direct effect	2.25 (1.44; 3.51)	> 99.9%	1.38 (0.86; 2.19)	91.8%

*Abbreviations:* Est. *OR*, Median of the marginal posterior distribution of adjusted odds ratios (OR), *CI* Compatibility interval (defined by the 2.5% and 97.5% percentiles of the posterior distribution), *Pr* Posterior probability

### Eight-week and 5-month prolonged abstinence

Estimates of natural direct and indirect effects on prolonged abstinence are presented in Table 4. The model including all three mediators shows that the effect of the intervention on 8-week abstinence was partially explained through the mediators measured at 1-month. For 5-month abstinence, the indirect effect of the mediators remained consistent, whilst the magnitude of the direct effect increased. Table 4 also details models for the single mediators, highlighting that know-how and confidence partially explained the effects of the intervention at both time intervals. Importance failed to explain any of the effects at both time intervals, which reflects the high level of importance reported by both groups at the start of the intervention.

### Four-Week point prevalence

Estimates of natural direct and natural indirect effects on 4-week point prevalence are presented in Table 5. The model including all three mediators show that the effect of the intervention on the 3-month outcome was partially explained by 1-month mediators. The 3-month mediators also partially explained the effects at 6 months. For the single mediators, know-how and confidence partially explained the effects at both time intervals, whilst importance failed to explain any of the intervention effects at both time intervals. Again, this reflects the high importance reported by both groups at the start of the intervention.

## Discussion

The current study investigated the mediated effects of a digital intervention on smoking abstinence. We found evidence that the effects of the intervention on smoking abstinence were partially mediated through knowing how to quit smoking and having confidence in being able to quit. Indirect effects partially explained the total effect on abstinence at both 3- and 6-months, with evidence of an additional direct effect at 6 months post-randomisation. This is consistent with the estimates of the effect of the intervention on mediators, whereby the groups diverged regarding know-how and confidence after the initial month, but the difference remained consistent over time for know-how and increased slightly for confidence. Initially, importance was rated highly for both groups and remained consistently high throughout the trial period, with no group differences being observed. The results suggest that those seeking help to quit smoking may benefit from guidance on how to build confidence in quitting and develop knowledge on how to enact cessation behaviours.

The current study highlighted that confidence in being able to quit smoking had a mediating effect. Confidence in this sense is reflective of self-efficacy, which has been shown to mediate the effects of other digital smoking cessation interventions [26–30]. Furthermore, Hoeppner et al. [28] reported that know-how also mediated the effects on smoking cessation. The current study supports these findings, however, Hoeppner et al. found that confidence in being able to quit smoking was a stronger mediator than know-how, whereas in our study we found that know-how was the strongest mediator. The difference in results for know-how between the trials may be resultant of the differing content: the intervention studied in Hoeppner et al. had a larger focus on enhancing motivation to stay abstinent compared to the intervention studied here, which had a stronger focus on developing the know-how on how to quit. Nonetheless, the replication of the results regarding know-how as a mediator is pertinent as this construct can be targeted by various self-change BCTs, that have been shown to be effective in eliciting smoking cessation [31, 32].

Theories that predict behaviour change for smoking cessation (e.g., stages of change or the health belief model, see Ravi et al. [33] for a review) suggest that reflective, associative, and self-regulation processes are key for sustaining behaviour change. The current study supports this suggestion, the mediating factors of know-how and confidence are underpinned by such processes. The behavioural techniques used to foster confidence and know-how, (e.g., distraction, tips to avoid craving or identification and avoidance of triggers) are either associative or self-regulatory in nature. This suggests that interventions aiming to sustain smoking abstinence may benefit from including techniques driven by associative and self-regulatory processes that aim to boost confidence and develop knowledge to quit smoking. Nonetheless, as this is a single trial, we would err on the side of caution in following this suggestion until further work can be carried out; however, evidence regarding behaviour change techniques (BCTs) used in smoking cessation trials highlights that associative and self-regulation techniques were the primary predictors of smoking cessation [11]. Furthermore, BCTs focusing on rewards (i.e., BCT taxonomy cluster 10, see Michie et al. [34] were particularly effective for written interventions (either in print or digital format) [11]. The rewards component used in the parent trial may have been mediated by confidence; hence, future digital interventions may also wish to include a reward component targeting confidence in being able to quit smoking, potentially focusing on the intrinsic (e.g., improved health, self-confidence) and extrinsic rewards (e.g., social approval, financial gains).

The results indicate that the total observed effect of the digital smoking cessation intervention was not fully explained by the mediators assessed in this study. Whilst this result may be explained by a limitation of capturing the extent of know-how, confidence, and importance using single-face items, it may be that other factors not included in the trial were mediating the effects. One such factor could be attitudes towards smoking cessation; positive attitudes towards smoking cessation have been shown to predict abstinence and quit attempts [31, 35]. It may be possible that increasing confidence and enabling the development of knowledge on how to quit smoking may have increased positive attitudes towards smoking cessation. This may be particularly applicable to the sample participants, who were generally older and had experienced failed quit attempts in the past—their attitudes towards cessation support may have been lower at the start of the trial and through the initial effect of the intervention, attitudes may have changed.

### Strengths and limitations

The study used time-lagged mediator measures, which ensures that the direction of causality in the mediator model is valid. Also, since data came from an RCT, we can consider the treatment-mediator and treatment-outcome relationships as unconfounded. However, the relationship between mediator and outcome is potentially confounded. When estimating this relationship, we adjusted for baseline characteristics to partially account for potential confounding, however, residual confounding may still exist, and this is a property of the model that cannot be tested. Findings could, therefore, be biased both away and towards the null. Attrition is also a major limitation of this study. The effectiveness trial, which enabled the enclosed mediator analyses, had minimal barriers to participation—participants found and signed up for the trial on their own. It is not surprising that attrition to follow-up subsequently was high. Attrition analyses did reveal that older participants were more likely to respond to follow-up in both groups and that those with higher baseline scores of nicotine dependence in the intervention group were more likely to respond to follow-up. However, the difference between groups was explained by nicotine dependence, and the models were adjusted for age and nicotine dependence at baseline. Analyses where missing data was imputed showed an attenuation towards the null for direct effects, but not so for indirect effects, and findings regarding mediation were not different. Anticipating high attrition, we decided to reduce participant burden and use three single-item face-valid measures for the mediator outcomes rather than empirically supported multi-item questionnaires. We should be cautious in our interpretation of findings because these measures have limited evidence of construct validity.

A further limitation is a lack of consideration for additional covariates, for example education level has been shown to impact cessation rates, with a higher probability of successful cessation amongst those with higher education [36]. Future studies should consider including additional factors associated with cessation. Finally, the study is limited due to the use of self-reported outcomes rather than, for instance, nicotine saliva tests. This limitation is exacerbated because participants were not blinded—they knew whether they received the digital intervention—and outcomes may be biased due to social desirability and compensatory rivalry effects. However, the effects of the intervention on the mediator measures and their subsequent influence on the outcome indicate that the estimated effects may not be entirely due to these biases. The intervention attempted to change the know-how and confidence of participants; this change was observed, leading to increased smoking abstinence. Of course, if the mediator measures themselves were affected by social desirability and compensatory rivalry effects then this pattern would also emerge; thus, we should see this as relevant to interpreting the risk of bias rather than conclusively ruling them out. It should be noted that even when biological verification is possible, it introduces its own biases, perhaps most prominently in the form of ascertainment bias.

## Conclusion

A digital smoking cessation intervention was found effective in prolonging self-reported smoking abstinence through improving know-how and confidence in participants' ability to quit smoking. The mediators did not fully explain the total effects, therefore, future studies should explore alternative mediators.

### Supplementary Information

Supplementary Material 1.

Supplementary Material 2.

## Data Availability

A study protocol, including a statistical analysis plan, is available open-access. Deidentified datasets generated during and/or analysed during the current study will be made available upon reasonable request to MB, after approval of a proposal and with a signed data access agreement.
