# Supplementary material for: Mediated effects of a randomised control trial for a text messaging smoking cessation intervention for online help-seekers and primary care visitors
Source: BMC Public Health. 2024 Jul 9;24:1824. doi: 10.1186/s12889-024-19273-4 (PMC11232307; doi:10.1186/s12889-024-19273-4)
Supplement: Supplementary file 1 — Supplementary Material 1. [file 12889_2024_19273_MOESM1_ESM.docx]

# Appendix A – Imputed data analysis

We used multiple imputation with chained equations to impute missing data for mediators at the 1-, 3- and 6-month follow-up intervals and smoking outcomes at the 3- and 6-month intervals. Baseline data was complete for all participants. We used the *mice* package (3.16.0) in R (version 4.3.1) with mediator variables, outcome variables, baseline variables, and group allocation as input.

Mediator variables were considered continuous, and we used predicative mean matching during the imputation process (smoking outcomes were binary). We generated 200 data sets using a maximum of 50 iterations for each generation. We then estimated each mediation model on each generated data set and collated the posterior distribution samples to create posterior distributions of each effect estimate with the uncertainty of missing data incorporated.

Supplementary Table 1 presents estimates of standardised effects of the intervention on mediator variables with missing data imputed. As pointed out in the main text, there was little difference between findings from the available data analyses and the imputed analyses. In Supplementary Tables 2 and 3, estimates of natural direct and indirect effects on prolonged abstinence and point prevalence of abstinence with missing data imputed are presented. Estimates of direct effects were attenuated towards the null in the imputed analyses, with indirect effects being similar to those estimated using available data in the main text. Overall, while direct effects were attenuated, interpretation of findings with imputed and available data were the same.

Supplementary Table 1 - Estimate of adjusted standardised effects of treatment on mediator factors at 1-, 3- and 6 months with imputed data

|  | **1-Month** | | **3-Month** | | **6-Month** | |
| --- | --- | --- | --- | --- | --- | --- |
|  | **Est. 95% CI** | **Pr. (Est. > 0)** | **Est. 95% CI** | **Pr. (Est. > 0)** | **Est. 95% CI** | **Pr. (Est. > 0)** |
| **Importance** | | | | | | |
| Intervention vs. Control | -0.07  (-0.24; 0.08) | 82.7% | 0.09  (-0.06; 0.25) | 87.8% | 0.05  (-0.12; 0.21) | 70.8% |
| **Know-how** | | | | | | |
| Intervention vs. Control | 0.51  (0.35; 0.66) | > 99.9% | 0.43  (0.26; 0.60) | > 99.9% | 0.42  (0.26; 0.58) | > 99.9% |
| **Confidence** | | | | | | |
| Intervention vs. Control | 0.23  (0.08; 0.38) | > 99.8% | 0.17  (0.00; 0.34) | 97.4% | 0.24  (0.07; 0.40) | 99.8% |
| **Abbreviations:** Est. – Median of the marginal posterior distribution of adjusted standardised effects CI – Compatibility interval (defined by the 2.5% and 97.5% percentiles of the posterior distribution) Pr. – Posterior probability | | | | | | |

Supplementary Table 2 – Estimates of natural direct and indirect effects on 8-week/5-month prolonged abstinence with imputed data

|  | **1-month mediator ->  3-month outcome** | | **3-month mediator ->  6-month outcome** | |
| --- | --- | --- | --- | --- |
|  | **Est. OR 95% CI** | **Pr. (OR > 1)** | **Est. OR 95% CI** | **Pr. (OR > 1)** |
| **Full model (All three mediators)** | | | | |
| Natural indirect effect | 1.52  (1.22; 1.95) | > 99.9% | 1.27  (1.08; 1.57) | 99.8% |
| Natural direct effect | 1.45  (0.98; 2.15) | 96.8% | 1.93  (1.3; 2.89) | 99.9% |
| **Importance** | | | | |
| Natural indirect effect | 0.98  (0.9; 1.03) | 82.5% | 1.04  (0.96; 1.16) | 87.7% |
| Natural direct effect | 2.03  (1.45; 2.86) | > 99.9% | 2.2  (1.53; 3.23) | > 99.9% |
| **Know-how** | | | | |
| Natural indirect effect | 1.43  (1.22; 1.73) | > 99.9% | 1.24  (1.1; 1.48) | > 99.9% |
| Natural direct effect | 1.46  (1.02; 2.11) | 98.0% | 1.86  (1.27; 2.75) | 99.9% |
| **Confidence** | | | | |
| Natural indirect effect | 1.25  (1.07; 1.49) | 99.8% | 1.12  (1.0; 1.3) | 97.4% |
| Natural direct effect | 1.67  (1.16; 2.41) | 99.7% | 2.07  (1.42; 3.05) | > 99.9% |
| **Abbreviations:** Est. OR – Median of the marginal posterior distribution of adjusted odds ratios (OR) CI – Compatibility interval (defined by the 2.5% and 97.5% percentiles of the posterior distribution) Pr. – Posterior probability | | | | |

Supplementary Table 3 – Estimates of natural direct and indirect effects on 4-week point prevalence with imputed data

|  | **1-month mediator ->  3-month outcome** | | **3-month mediator ->  6-month outcome** | |
| --- | --- | --- | --- | --- |
|  | **Est. OR 95% CI** | **Pr. (OR > 1)** | **Est. OR 95% CI** | **Pr. (OR > 1)** |
| **Full model (All three mediators)** | | | | |
| Natural indirect effect | 1.33  (1.11; 1.61) | 99.9% | 1.31  (1.11; 1.6) | 99.9% |
| Natural direct effect | 1.31  (0.9; 1.9) | 91.9% | 1.31  (0.9; 1.89) | 92.1% |
| **Importance** | | | | |
| Natural indirect effect | 0.98 (0.9; 1.02) | 82.3% | 1.04  (0.97; 1.16) | 87.8% |
| Natural direct effect | 1.7  (1.2; 2.4) | 99.9% | 1.56  (1.09; 2.21) | 99.3% |
| **Know-how** | | | | |
| Natural indirect effect | 1.31  (1.14; 1.55) | > 99.9% | 1.28  (1.13; 1.52) | > 99.9% |
| Natural direct effect | 1.3  (0.9; 1.87) | 92.1% | 1.29  (0.9; 1.85) | 91.9% |
| **Confidence** | | | | |
| Natural indirect effect | 1.15  (1.05; 1.3) | 99.8% | 1.12  (1.0; 1.29) | 97.4% |
| Natural direct effect | 1.45  (1.02; 2.07) | 98.0% | 1.45  (1.02; 2.09) | 98.0% |
| **Abbreviations:** Est. OR – Median of the marginal posterior distribution of adjusted odds ratios (OR) CI – Compatibility interval (defined by the 2.5% and 97.5% percentiles of the posterior distribution) Pr. – Posterior probability | | | | |
