# Supplementary material for: Mediated effects of a randomised control trial for a text messaging smoking cessation intervention for online help-seekers and primary care visitors
Source: BMC Public Health. 2024 Jul 9;24:1824. doi: 10.1186/s12889-024-19273-4 (PMC11232307; doi:10.1186/s12889-024-19273-4)
Supplement: Supplementary file 2 — Supplementary Material 2. [file 12889_2024_19273_MOESM2_ESM.docx]

# Appendix B – Attrition analyses

## Baseline characteristics and non-response

There was evidence that older individuals in both groups were more likely to respond to follow-up of mediator variables at all three intervals. At the 1-month interval, the odds ratio (OR) of non-response was 0.97 (95% CI = 0.96; 0.98, posterior probability of association [POA] > 99.9%); at the 3-month interval the OR was 0.99 (95% CI = 0.98; 1.00, POA = 98.4%); and at the 6-month interval the OR was 0.98 (95% CI = 0.97; 0.99, POA > 99.9%). Supplementary Figure 1 a-c visualizes the relationship between missingness at the follow-up intervals and age.

|  |  |
| --- | --- |
| (a) | (b) |
|  |  |
| (c) |  |

Supplementary Figure 1 – Associations between age and missingness of mediator data at the 1-month (a), 3-month (b), and 6-month (c) follow-up intervals.

There was also evidence that among intervention group participants only, those who scored higher on the Fagerström test for nicotine dependence at baseline were more likely to not respond at the 1-month follow-up interval. However, this association was not found at the subsequent follow-ups at 3- and 6-months. Further, this association explained the differential follow-up rates between groups entirely, as group was associated with an OR of 1.0 (0.92; 1.13, POA = 52.4%) in the interaction model, yet the group by Fagerström test for nicotine dependence score was associated with an OR of 1.08 (1.0; 1.2, POA = 95.5%) and no other interaction terms were markedly associated with missingness. Supplementary Figure 2 a-c visualizes the relationship between missingness at the follow-up intervals and age.

|  |  |
| --- | --- |
| (a) | (b) |
|  |  |
| (c) |  |

Supplementary Figure 2 - Associations between baseline scores of Fagerström test nicotine dependence test and missingness of mediator data at the 1-month (a), 3-month (b), and 6-month (c) follow-up intervals.

## Total effect estimates

The estimates of total effect of the intervention on smoking abstinence from the trial’s original primary analyses and those estimated using data available for the mediation analyses are shown in Supplementary Table 4. There were marked inflation of effects at the 3-month follow-up interval for both outcomes. This was also evident from direct effects being smaller when using imputed data (see Appendix A). However, overall, these differences in effect estimates did not affect our interpretation of findings regarding indirect effects.

Supplementary Table 4 - Estimates of total effect on smoking abstinence outcomes from the trial’s original primary analyses and using data available for mediation analyses.

|  | **3-month follow-up interval** | | **6-month follow-up interval** | |
| --- | --- | --- | --- | --- |
|  | **Est. OR 95% CI** | **Pr. (OR > 1)** | **Est. OR 95% CI** | **Pr. (OR > 1)** |
| **8-week / 5-month prolonged smoking abstinence** | | | | |
| Original | 2.15 (1.51; 3.06) | > 99.9% | 2.38 (1.62; 3.57) | > 99.9% |
| Mediation | 3.05 (1.96; 4.80) | > 99.9% | 2.96 (1.83; 4.78) | > 99.9% |
| **4-week point prevalence of smoking abstinence** | | | | |
| Original | 1.70 (1.18; 2.44) | 99.8% | 1.49 (1.03; 2.14) | 98.3% |
| Mediation | 2.77 (1.81; 4.33) | > 99.9% | 1.51 (0.96; 2.39) | 96.3% |
| **Abbreviations:** Est. OR – Median of the marginal posterior distribution of adjusted odds ratios (OR) CI – Compatibility interval (defined by the 2.5% and 97.5% percentiles of the posterior distribution) Pr. – Posterior probability | | | | |
